# Proteomics-Based Analysis of Protein Complexes in Pluripotent Stem Cells and Cancer Biology 

**DOI:** 10.3390/ijms17030432

**Published:** 2016-03-22

**Authors:** Putty-Reddy Sudhir, Chung-Hsuan Chen

**Affiliations:** Genomics Research Center, Academia Sinica, Taipei 11529, Taiwan; preddy@gate.sinica.edu.tw

**Keywords:** protein complexes, affinity purification, mass spectrometry, proteomics, pluripotent stem cells, cancer biology

## Abstract

A protein complex consists of two or more proteins that are linked together through protein–protein interactions. The proteins show stable/transient and direct/indirect interactions within the protein complex or between the protein complexes. Protein complexes are involved in regulation of most of the cellular processes and molecular functions. The delineation of protein complexes is important to expand our knowledge on proteins functional roles in physiological and pathological conditions. The genetic yeast-2-hybrid method has been extensively used to characterize protein-protein interactions. Alternatively, a biochemical-based affinity purification coupled with mass spectrometry (AP-MS) approach has been widely used to characterize the protein complexes. In the AP-MS method, a protein complex of a target protein of interest is purified using a specific antibody or an affinity tag (e.g., DYKDDDDK peptide (FLAG) and polyhistidine (His)) and is subsequently analyzed by means of MS. Tandem affinity purification, a two-step purification system, coupled with MS has been widely used mainly to reduce the contaminants. We review here a general principle for AP-MS-based characterization of protein complexes and we explore several protein complexes identified in pluripotent stem cell biology and cancer biology as examples.

## 1. Introduction

Proteins often interact and form protein complexes with nucleic acids (*i.e.*, DNA or RNA), proteins, peptides, small molecules, or metal ions [1,2]. Here, we mainly focus on protein complexes that comprise protein-protein interactions as they play major roles in diverse molecular functions and cellular processes. The protein-protein interactions occur in the extracellular space, inside the cells, and in the region of the cell membrane. Identification of protein complexes is important as we can define the roles of individual proteins of interest in association with their interaction partners [3]. The protein-protein interactions have been widely identified by means of yeast-2-hybrid (Y2H) as well as by affinity purification coupled with mass spectrometry (AP-MS) [4,5]. On the other hand, structural information of the protein complexes have been studied using chemical cross-linking or hydrogen-deuterium exchange coupled with MS analysis [6]. The chemical cross-linking approach is useful to provide distance of proteins within a protein complex and the hydrogen-deuterium exchange approach is useful to identify the protein binding sites.

The basic principle of the Y2H approach, a genetic and *in vivo* assay, was first described by Fields and Song in 1989 to study direct, binary protein-protein interactions [7]. In general, the Y2H method involves transcription activation and reporter gene expression. Transcription factors consists of a DNA binding domain (DBD) and transcription activation domain (TAD), which mediate DNA binding to the promotor region and RNA polymerase machinery interactions, respectively. First, the plasmids of chimeric proteins were constructed by fusing the DBD and TAD domains with bait and prey proteins respectively. Further, these two chimeric proteins were expressed in the yeast system and their interaction resulted in the reformation of active transcription factor. The active transcription factor mediates the expression of the corresponding reporter gene that indicates the protein (bait)-protein (prey) interaction. The Y2H system is able to detect both transient and stable protein-protein interactions via the expression of a reporter gene [8]. The other advantage is that drug compounds inhibiting binary protein-protein interactions can be identified by the Y2H system [9].

Despite the advantages, there are a few drawbacks of the Y2H system. For example, autoactivation of the transcription by bait protein in absence of prey protein and subsequent reporter gene expression results in identification of false positive protein interactions [8]. On the other hand, the Y2H system may also give false negative results because of the mammalian cell environment, but not the yeast cell system, maintains proper protein folding, protein localization, protein activation, signaling, and post-translational modifications, which are often required for protein-protein interactions [8,10]. The other limitation of Y2H is that expression of a bait/prey protein may decrease yeast growth due to its toxic nature [8,11]. A major concern is that the Y2H approach mostly detects binary interactions: the Y2H approach is laborious and time-consuming to elucidate an interactome of a bait (or target protein) of interest [8]. Another method known as AP-MS has been used to solve this problem. In affinity purification, an endogenous target protein or an ectopically expressed affinity-tagged target protein is used as a bait to purify its protein complex. Subsequent MS analysis of the purified complex results in identification of an interactome of the target protein. Here, we review the affinity purification approach, MS analysis of protein complexes, the quantitative AP-MS approach, protein interaction databases, and protein complexes associated with physiological (e.g., stem cell pluripotency) and pathological (e.g., cancer) conditions.

## 2. Affinity Purification of Protein Complexes

**Figure 1:**
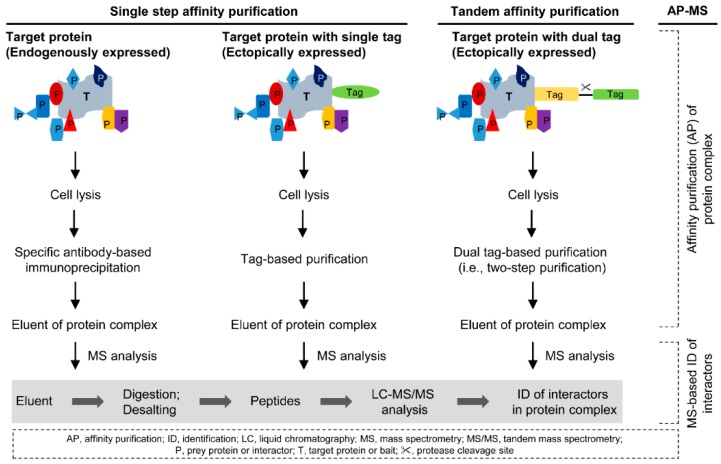
Schematic presentation of the general principle of affinity purification coupled with mass spectrometry (AP-MS) approach. The outlines of single step and tandem affinity purification of protein complex are shown. The purification process is performed once and twice, respectively, in single step and tandem affinity purification approaches. The eluent of the protein complex is subjected to enzymatic digestion and subsequent MS analysis to identify the interactors of the target protein. The sizes of target and prey proteins do not reflect their molecular weights. The different colors/shapes indicate different prey proteins or tags. The gray color box highlights the MS analysis part of AP-MS approach. The figure is drawn based on the concepts described in multiple studies [3,4,12,13,14,15,16].

Two well-established affinity purification approaches are available. They are single step affinity purification and tandem affinity purification (TAP) [12,13,14]. The outlines of purification methodology are shown in detail in Figure 1.

### 2.1. Single Step Affinity Purification

Single step affinity purification can be divided into two types based on the endogenously expressed and ectopically expressed target proteins. The first type of single step affinity purification is popularly known as immunoprecipitation (IP). In this approach, the endogenously expressed target protein along with its interaction partners is purified using a specific antibody and a matrix such as protein A/G beads (Figure 1, left panel) [17]. This approach is applicable for both cell lines and tissue samples. However, cross-reactivity of the antibody is a major problem associated with the IP procedure [3,13]. The second type of single step affinity purification (Figure 1, middle panel) has been used when endogenous expression of target protein is low or the specific antibody of target protein is not available. In this approach, a selected affinity tag is fused with the target protein to produce the recombinant protein (*i.e.*, affinity-tagged protein). The affinity captured target protein is subjected to stringent washing to remove possible nonspecific protein interactions, and then purified by using elution buffer. The affinity purified sample is digested and desalted ahead of MS-based identification of interaction partners of target protein (Figure 1). However, stringent purification may lead to the loss of specific protein interactions that are weakly associated with the target protein. Recently, an approach known as affinity enrichment mass spectrometry (AE-MS), which directs low stringent purification, has been reported to identify the protein interactions [18]. This method utilized different tagged target proteins rather than a single tag-less control to distinguish the specific interacting proteins. Another recent study has utilized multiple affinity resins and performed parallel affinity purification of a doubly tagged target protein to discriminate nonspecific interactions from specific interactions [19].

A variety of tags are available for affinity purification and the most popular tags are DYKDDDDK peptide (FLAG), glutathione-*S*-transferase (GST), hemagglutinin (HA), polyhistidine (His), maltose-binding protein (MBP), Myc, and streptavidin (Strep) [12,13,14,20]. The tagging has been suggested both at C and N-terminal regions of the target protein based on the protein function and protein interaction characteristics [4,21]. The affinity-tagged protein is expressed in live cells and then purified along with its interaction partners by using tag-specific antibody or tag-specific matrix. Therefore tag-based affinity purification is not applicable for clinical samples (*i.e.*, healthy and diseased tissues). The purified complexes are generally eluted by changing the pH or salt condition, or by binding competition with a tag-specific matrix based on the experimental conditions [12,13,14].

Here we describe two examples of tags (FLAG and His) used in single step affinity purification [12]. The FLAG tag is a small (*M*_W_ of 1012.98 kDa) hydrophilic peptide with amino acid sequence DYKDDDDK. The FLAG-tagged target protein along with its interacting partners is purified by using anti-FLAG antibody specific to the FLAG epitope. The competitive elution could be performed using FLAG peptide. The FLAG tag can be removed from the purified target protein using the specific protease, enterokinase for further use of the target protein (e.g., structural analysis or functional activity analysis) [21]. On the other hand, the His tag, which contains 5–10 histidine repeats, is also widely used to purify the protein complexes. The His-tagged protein along with its interacting partners is purified using immobilized metal (Co, Cu, Ni, or Zn) ions, which can be selected based on experimental conditions. The protein complex of His-tagged target protein will be eluted usually by using imidazole. Although the His tag rarely affects the protein activity, it may be significant to remove it by introducing a protease cleavage site between the tag and target protein. Designing novel probes or tags for affinity purification is critical for efficient identification of the interactomes of target proteins. For example, a recent study has synthesized a probe to specifically enrich several Src homology 2 (SH2) domain containing proteins by inhibitor affinity purification approach [22]. In another example, affinity probes have been designed and validated to isolate the Hsp70 complexes [23].

### 2.2. Tandem Affinity Purification

The methodological development of single step tag-based purification resulted in an approach known as tandem affinity purification (TAP) [16]. This approach requires fusion of target protein to a tandem tag (TAP tag) with a protease cleavage site located between the two tags (Figure 1, right panel). The fusion construct is then expressed in host cells to purify the protein complex of the target protein. The TAP process can be divided into two major steps [3,14,16]. The first step involves capture of the protein complex using tag-specific matrix/antibody, washing, and elution by protease cleavage. The second step involves capture of the protein complex again using a tag-specific matrix/antibody, washing, and elution. This two-step purification, which involves two washings, not only reduces the contaminants but may also lead to the loss of weakly interacting proteins. The demonstrated TAP tag (*M*_W_ of 20.7 kDa) is a combination of calmodulin-binding peptide (CBP) and IgG-binding protein A tags, which were separated by a TEV (tobacco etch virus) protease cleavage site [16]. This is because the authors tested various tags including FLAG, His, Strep, IgG-binding Protein A, calmodulin-binding peptide (CBP), and chitin-binding domain (CBD) and observed the best results when they used the fusion protein with CBP and IgG-binding Protein A tags [16]. However, the demonstrated TAP tag is not acceptable for mammalian cells as the endogenous calmodulin and calmodulin-binding proteins affect the purification process [24,25]. Burckstummer *et al.* [24] have showed GS-TAP tag (*M*_W_ 18.8 kDa), which is a combination of protein G and streptavidin-binding peptide (SBP) tags, with 10-fold increase in yield when compared with demonstrated TAP procedure. Tagging proteins with high molecular weight tags may affect the proper folding ability, functional activity, and binding capacity [3,26]. Therefore TAP tags such as FLAG-HA, 3X FLAG-His, 2X Strep II-FLAG, and SBP-HA with molecular weights about 5 kDa have been established [25,26]. In addition, a variety of TAP tags and their advantages were described elsewhere [25].

The original TAP approach has been demonstrated in the yeast system [16]. The yeast system allows integration of a tag of the target gene into the genome by a homologous recombination process [27,28]. This process allows the expression of tagged version of the target protein only and bypasses the expression of untagged endogenous target protein. In contrast, the tagged version of target protein as well as untagged endogenous target protein are expressed simultaneously in higher eukaryotic cells. Thus, the endogenous target protein may interfere with the purification process [29]. To bypass such interference, an approach combining RNAi and TAP system has been demonstrated, which depletes the endogenous target protein [29]. The other problem is that handling full-length cDNAs of large transcripts is challenging in mammalian systems. In addition, cDNA-based transgene expression does not resemble the endogenous system. To overcome these problems, BAC (bacterial artificial chromosomes) transgenes have been used by Poser and coworkers [30]. These transgenes consist of most regulatory elements and their stable transfection into host cells may mimic the endogenous gene expression. Therefore BAC transgenes have been recommended to study the protein complexes by AP-MS [30,31]. Poser *et al.* [30] have demonstrated the application of BAC transgenes to study the protein localization, protein-protein interactions, and protein-DNA interactions by using tag-based assays. Further, an application of quantitative proteomics approach combined with TAP has been shown to identify the low abundance proteins associated with the target protein [32]. The AP-MS approach using soluble fractions of whole cell extracts may bypass the target protein interactions that occur in nonsoluble fractions (e.g., chromatin). A method known as multiple cell compartment AP-MS/MS (MCC-AP-MS/MS), which is based on fractionation, has been introduced to identify the compartment specific protein interactions [33].

## 3. Basics of Mass Spectrometry (MS) Analysis

### 3.1. Sample Preparation and MS Analysis

The next step is identification of interactors within the purified protein complex by MS analysis (Figure 1). Although coupling affinity purification with co-immunoprecipitation (CoIP) is the conventional method to characterize the interactors, it is a tedious process when compared with MS analysis, which allows identification of all interactors in a single or replicate LC-MS/MS runs. For MS analysis, sample preparation is critical and several studies have described the standard enzyme digestion procedures [15,34,35,36,37,38,39,40,41]. Two well-defined strategies are available for sample preparation: in-gel and in-solution digestion. With the in-gel digestion approach [15,35], the purified samples were denatured in sample buffer and separated by sodium dodecyl sulfate-polyacrylamide gel electrophoresis (SDS-PAGE) analysis. Then protein bands were visualized by silver staining or Coomassie blue staining as per protein concentration. The gel is fractionated and each fraction is sliced into small pieces. The proteins present in small gel pieces were generally digested with trypsin. Alternatively, tube-gel digestion [34,37] or in-solution digestion [36,38,40] without fractionation is applicable for those purified samples with less complexity. The tube-gel digestion does not require running SDS-PAGE. In tube-gel digestion, the protein sample is mixed with acrylamide in an Eppendorf tube and then the solidified gel matrix is sliced into small pieces to perform trypsin-digestion. However, complete peptide extraction from the gel matrix is difficult and thus sample loss may occur during gel-based digestion processes. In the in-solution digestion approach, denaturation is performed in the presence of urea with higher concentration, dithiothreitol (DTT), and iodoacetoamide (IAA). Once the denaturation process is completed, the urea concentration is reduced to perform optimal trypsin digestion. Recently, filter aided sample preparation (FASP) approach with the advantages of both in-gel and in-solution digestion approaches has been demonstrated [39,41]. The prepared samples from the above methods were then subjected to MS analysis.

Although a variety of commercial mass spectrometers are available, the basic principle is similar for MS-based protein analysis. We recommend to readers other excellent reviews on basic principles of MS instrumentation [42,43,44], which is beyond the scope of this review. Briefly, MS mainly consists of the ion source, a mass analyzer, and an ion detector [15,42,43,44]. The peptides can be ionized by two strategies: electrospray ionization (ESI) or matrix-assisted laser desorption/ionization (MALDI). In the MALDI approach, the digested samples were crystalized on a target plate by mixing with a suitable matrix solution. The crystals were then exposed to laser pulses to desorb and ionize the peptides [15,42,43]. In the ESI approach, the digested samples were generally mixed with an acidic solution and sprayed as droplets by means of a nebulization gas [42,43,44]. The desolvation gas (*i.e.*, sheath gas) assists in droplet evaporation and ejection of peptide ions at high voltage. The peptide ions were then transferred to the mass analyzer (e.g., time-of-flight (TOF), orbitrap, ion trap, or hybrid type) that is placed in high vacuum. The analyzed ions were subsequently recorded as mass spectra by the ion detector. The raw data obtained from the MALDI- or ESI-MS instrument is searched against the database and proteins of the protein complexes were identified [43]. ESI coupled with liquid chromatography (LC)-MS/MS is recommended for characterization of interactors of protein complexes. This is because of the ease of integrating ESI-MS with the LC system, which is critical for peptide separation [43]. In addition, recent developments in hybrid type mass analyzers made ESI-LC-MS/MS as efficient as possible for the analysis of peptides with high resolution, high accuracy, and high sensitivity.

### 3.2. MS-Based Label-Free Quantitative Analysis

In addition to the identification of interactors within the protein complex, their relative expression levels can be quantified between two or more biological conditions by using quantitative MS analysis. Two types of MS-based quantitative approaches are available: labeling and label-free methods. Labeling can be performed at three levels to achieve accurate protein quantitation [4,45]. Metabolic labeling of proteins, which occur at the cellular level, can be achieved by SILAC (stable isotope labeling by amino acids in cell culture) approach. Protein or peptide level labeling (e.g., ICAT (isotope-coded affinity tag) approach) and peptide level labeling (e.g., iTRAQ (isobaric tags for relative and absolute quantitation), TMT (tandem mass tag), ^18^O, ICAT approaches) can be performed by using the commercially available labeling reagents. However, recent advances in MS analysis and software development for data analysis has made the label-free quantitation (LQ) a powerful technique in quantitative proteomics [46]. The LQ is cost-effective when compared with labeling methods and is applicable for both cell cultures and tissue samples. In addition, protein quantitation can be performed across a large number (theoretically unlimited) of biological samples/conditions. The LQ depends on signal-to-noise ratio, retention time, charge state, and isotopic patterns of the detected peptide peaks.

**Figure 2:**
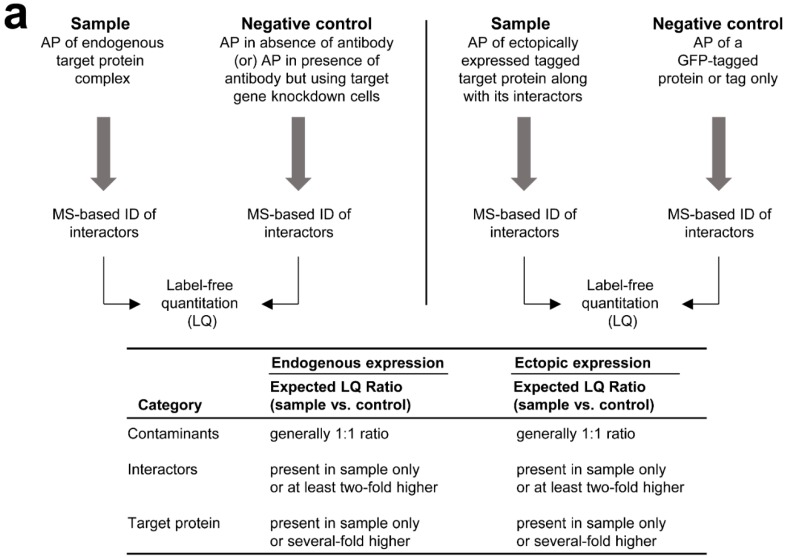

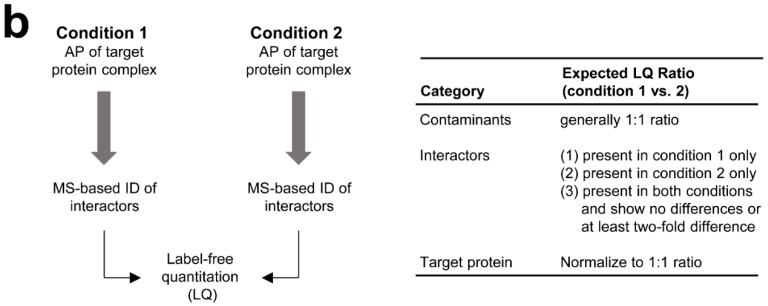
Application of MS-based label-free quantitation (LQ) to study protein complexes. (**a**) The MS-based LQ to identify the contaminants or non-specific interactors is shown. The protein complex eluent of endogenous (**left panel**) or ectopically (**right panel**) expressed target protein is collected by affinity purification (AP). In addition, the corresponding eluent of negative control is collected by means of AP. The eluents are subjected to enzymatic digestion and subsequent MS analysis (Figure 1). The MS data of sample and negative control are compared by LQ and the anticipated results are shown in the bottom panel; (**b**) The MS-based LQ to identify the differential protein interactions is shown. The protein complex of endogenous or ectopically expressed target protein is eluted from two biological conditions by means of AP. It is important to maintain the negative controls as described in Figure 2a. The eluents are subjected to enzymatic digestion and subsequent MS analysis (Figure 1). The MS data obtained from conditions 1 and 2 are compared by LQ and the anticipated results are shown in the right panel. The figure is drawn based on the concepts described in multiple studies [4,20,31,45,47,48,49,50,51].

Labeling and label-free quantitation approaches have been widely used for the identification of contaminants within the protein complex [13,20,31]. In the case of endogenous target protein (Figure 2a, left panel), protein complex is captured by a specific antibody bound to the protein G/A beads. Simultaneously, the protein G/A beads without a specific antibody can be used to maintain a negative control. The eluents obtained from both sample and negative control were subjected to digestion and MS analysis as depicted in Figure 1. The MS data corresponding to the sample and negative control were then compared by LQ to identify the specific interactors and non-specific contaminants. Alternatively, high confidence protein interactions within a protein complex can be achieved by QUICK (quantitative IP combined with knockdown) approach [47]. In the QUICK approach, the cells without (sample) and with (negative control) knockdown of a target gene in combination with labeling were used to immunoprecipitate the target protein. However, it is possible to perform the QUICK method by label-free approach as well. The anticipated LQ results for the above two approaches are shown in Figure 2a, bottom panel. In general, the contaminants show a ratio close to 1:1 between sample and negative control. The interactors show at least a two-fold higher ratio and the target protein shows a several-fold higher ratio in the sample compared to the negative control. Sometimes, the interactors and target protein were not comparable because they were identified in the sample only but not in the negative control. A similar approach has been used in the case of ectopically expressed tagged protein (Figure 2a, right panel). However, the negative control is maintained by expressing the tag only or green fluorescent protein (GFP)-tagged protein. The anticipated LQ results are similar to endogenous target protein purification (Figure 2a, bottom panel). Several studies, which used labeling and label-free approaches to identify the nonspecific interactions, are listed in a recent study [13]. In addition, a recent AP-MS study shown the application of SWATH (sequential window acquisition of all theoretical spectra) technology to identify the specific interactions of the bait protein by comparing the tagged-proteins (EIF4A2 and MEPCE) and GFP-tagged control [48].

In addition, labeling or label-free quantitation is useful for comparison of interactors of the protein complex between two or more biological conditions. In label-free approach (Figure 2b, right panel), the protein complexes of endogenous target protein or tagged target protein and their negative controls (controls not shown in figure) were purified under different biological conditions. The purified samples were subjected to MS analysis (Figure 2) by preparing the samples as shown in Figure 1, bottom panel. The samples were then compared by using their corresponding MS data. The anticipated results are shown in Figure 2b, left panel. Here, we can observe three types of interactors: the condition 1-specific, condition 2-speciifc, and shared interactors. In general, the target protein levels need to be normalized to a 1:1 ratio to compare the expression levels of shared interactors between the biological conditions. The examples include cancer-associated studies with labeling approaches, which demonstrated the quantitative interactomes of two splice variants of a gene [49] and mutant and wild-type forms of a gene [50]. In another example, the application of AP-MS combined with SWATH technology has been shown to quantify the protein interaction dynamics of 14-3-3 system under different conditions [51]. In addition, Fabre *et al.* [52] have shown the dynamics of proteasome complexes composition and stoichiometry by using multiple cell lines, affinity purification, and label-free quantitative proteomics. Furthermore, a recent study has developed a novel method to characterize the preferential interactions with in the protein complexes by using proteasome case as an example [53]. In another study, Fabre *et al.* [54] have compared different label-free quantitative approaches to verify the stoichiometry of the subunits of protein complexes. We refer to the readers a recent review for more details on quantitative AP-MS approaches to study the dynamics and subunits stoichiometry of protein interactions [55].

## 4. Resource Databases of Protein Interactions

The improvements in purification methods and developments in MS analysis tremendously increased the information related to protein interactions and their protein complexes. The experimental data related to protein complexes has been stored in a database known as the comprehensive resource of mammalian protein complexes (CORUM) [56,57]. In addition, several databases, including Biomolecular Interaction Network Database (BIND) [58], Database of Interacting Proteins (DIP) [59], Human Protein Reference Database (HPRD) [60], Human Protein Interaction Database (HPID) [61], an open source molecular interaction database (IntAct) [62], Molecular Interactions Database (MINT) [62,63], and Search Tool for the Retrieval of Interacting Genes/Proteins (STRING) [64,65] have collected the information on protein interactions [13]. These databases collect the information based on multiple approaches such as literature search of *in vitro*/*in vivo* experimental data and/or *in silico*-based predictions [5]. The protein interaction databases provide several advantages. For example, by using STRING database, one can search the known and predicted interaction partners of a target protein of interest in a specific organism. In addition, the STRING database provides an option to search the information on interactions of a list of target proteins including their interaction partners. The other example is that interaction partners of the protein complexes identified by various approaches including AP-MS analysis can be confirmed by verifying in protein interaction databases.

Computational approaches are necessary to extract the biological significance by systematic analysis of information stored in the protein interaction databases. The data obtained from protein interaction databases could be analyzed and comprehended by using a variety of tools such as Cytoscape [5,13]. For example, a study specifically showed the protein complexes related to human genetic disorders to explore their role in pathogenesis by using the information available in the protein interaction databases [66]. The other example is that proteomics data have been integrated with protein interaction databases to extract the protein complexes associated with diverse cellular functions [38,67,68].

## 5. The Examples of Protein Complexes Identified in Pluripotent Stem Cell Biology

The main types of pluripotent stem cells are embryonic stem cells (ESCs), and ESCs derived by somatic cell nuclear transfer (NT-ESCs), and induced pluripotent stem cells (iPSCs) generated by somatic cell reprograming [69,70,71,72]. Several genes (e.g., Oct4, Sox2, and Nanog) are identified as essential to maintain the stem cell pluripotency [69,70]. In recent years, great progress has been made in studying the interactomes of pluripotency-associated genes to understand the biology of pluripotent cells [73]. Especially, the interactomes of several transcription factors including Oct4, Sox2, Nanog, and c-Myc have been well-studied. Two studies have identified the Oct4 interactome in mouse ESCs by using the AP-MS approach that expanded our understanding of Oct4 roles in pluripotency, development, and disease [74,75]. These two studies have identified a total of 126 Oct4 interacting proteins, of which 20 were identified in both studies. Some of the identified Oct4 interacting proteins (e.g., Chd4, Hdac1, Lsd1, Mta2, Myst2, Parp1, and Sall4) were further confirmed by means of immunoblotting. An improved AP-MS method has been demonstrated [76] and identified 198 Oct4-associated proteins, of which 155 were not identified in previous studies [74,75]. By using CoIP/IP, the Oct4 interactions with Ash2l, Kif11, Msh2/6, Ppp1cc, Ring1B/Rnf2, Supt16h, and Zmym2 have been confirmed. The Oct4 interactors are mainly transcription factors or the components of chromatin remodeling complexes (SWI/SNF, ISWI, NuRD, and INO80), which are associated with pluripotency and cell reprograming. The interactome of Sox2 has been identified in mouse ESCs by using FLAG-Strep tag system and MudPIT [77]. This study revealed that Sox2 interactome consists of more than 70 proteins and also showed that one of the Sox2-associated proteins, Smarcd1 is required for ESC self-renewal and pluripotency. The Sox2 complexes with Klf4, Nanog, Oct4, and Rpa1 proteins have been validated. The interactome of Nanog has been identified in mouse ESCs using the AP-MS approach and is linked with transcription repression to regulate the gene expression and ES cell fate [78,79]. The interactomes of exogenously and endogenously expressed Nango were identified by using affinity purification. Each of two studies identified interactome with 17 proteins and showed an overlap of four proteins. Several of the Nanog interacting proteins (e.g., Dax1, Mta1, Mta2, Nac1, Oct4, Rif1, Sin3A, and Zfp281) were further confirmed by additional experiments. In addition, Nanog interaction network was further defined by using AP-MS-based identification of the interacting proteins of Nanog-associated proteins (Dax1, Nac1, Zfp281, and Oct4) and a stem cell marker, Rex1 [78]. The protein-protein and protein-DNA interactions of c-Myc have been explored and their combination revealed three (Core, Polycomb, and Myc) functional modules in ES cells [80]. Interestingly, Myc module is associated with transcription programs of both ES cells and cancer cells. An informatics-assisted integrative analysis is critical to extract the biological significance of the AP-MS-based proteomics studies as described in last paragraph of this section.

In addition to the above described protein interactions of core pluripotency genes, a recent study has demonstrated an interactome of RNA binding protein, L1TD1 (LINE-1 type transposase domain-containing protein 1) in human ESCs. The high levels of L1TD1 has been identified in ESCs and is involved in maintenance of human ES cells pluripotency. The AP-MS approach identified 306 proteins in the interactome of L1TD1 and several of them were known to be shared with Oct4, Sox2 and Nanog interactomes [81]. This study validated several of the identified interactors of L1TD1 including CTNNB1, DDX3X, DHX9, ELAVL1, IGF2BP1, IGF2BP3, KHDRBS1, KPNA2, PABPC1, and PARP1. According to the protein localization analysis, majority of the L1TD1 interactors are cytoplasmic (54%) and nuclear (32%) proteins, which signifies its functional role in both cytoplasm and nucleus. A classification based on protein structural domains revealed that 19% of the L1TD1 interactors are ribosomal proteins, suggesting that L1TD1 is associated with translation. In addition, ES-specific BAF complex has been identified by a proteomics approach, which showed the critical role of BAF complex in ES cell self-renewal and pluripotency [82]. The authors have identified BAF interactomes in mouse ES cells, embryonic fibroblasts (MEFs), and P0 brain cells and observed that the composition of BAF complex is significantly different in each cell type. The BAF interactome consists of 197 (ES cells), 112 (MEFs), and 58 (P0 brain) proteins based on ProteinProphet probabilities exceeding 95%. In particular, it has been confirmed that the BAF interactome consists of an association between Brg and BAF155 proteins, but not between Brg and BAF170, in ES cells when compared with the other two types of cells. This indicates the condition-specific interactome analysis of a target protein is critical for understanding the biology of a cell type of interest. The protein complexes identified in ES cells not only improves our knowledge on pluripotency biology but also serves as a baseline to improve the somatic cell reprograming efficiency.

To gain further insight into pluripotency, the protein interaction data of Oct4 and Nanog from the above mentioned four studies [74,75,78,79] was integrated into a single network comprising 239 proteins [83]. Of the 239, 131 proteins showed direct interactions with Oct3/4, suggesting that pluripotency is maintained by a large number of individual components in combination with core pluripotency genes. Another study further expanded the interactome of Nango, which not only revealed a direct interaction between Nanog and Sox2 but also identified a large number of Nanog interacting proteins that include mainly transcription factors and the components of chromatin remodeling complexes [84]. Furthermore, a protein-protein interaction landscape has been shown by integrating the Sox2 interactors and the interactomes of nine proteins including Dax1, Esrrb, Oct4, Nac1, Nanog, Rex1, Sall4, Tcfcp2l1, and Zfp28 identified in ESCs. This landscape consists of 334 proteins, indicating that these ten proteins are highly dependent on each other in a complex manner to maintain the pluripotency of ESCs [77]. Moreover, protein complexes have been identified by integrating the quantitative proteomics data of human ESCs, iPSCs, and parental cells with the information available in protein interaction databases such as CORUM and STRING [38]. The identified protein complexes are associated with chromatin remodeling, transcription regulation, RNA splicing, gene expression, RNA catabolism, DNA replication, DNA repair, cell cycle, metabolism and other cellular processes. Several of these identified protein complexes have been linked with pluripotency maintenance and embryonic development.

## 6. The Examples of Protein Complexes Identified in Cancer Cell Biology

Identification of disease-associated genes (e.g., cancer-associated genes) is critical for drug development, diagnosis, and therapy. The genes present in cancers but absent or expressed at lower levels in their corresponding healthy normal samples are known as cancer-associated genes. Typically, the cancer-associated genes/proteins were identified by conventional methods or omics approaches by looking into the fold-changes of gene expression levels between cancer and normal samples. However, the cancer-associated proteins interact with other proteins to initiate oncogenic signal transduction, which deregulates normal cellular functions and leads to cancer pathogenesis. Therefore, studying the protein complexes in cancers is important to understand the mechanisms associated with cancer pathogenesis.

Here, we attempt to highlight some of the receptor protein complexes identified in cancer cells. Epidermal growth factor receptor (EGFR) is a well-known oncogene that plays a key role in multiple cancers. A recent study characterized its interactome in epidermal carcinoma cells by AP-MS analysis [85]. This study identified the EGFR interactome with 183 proteins. Network analysis revealed that EGFR directly interacts with 15 of 183 proteins, including Grb2, shc-1, and STAT1/3. This study has verified the EGFR interaction with AP2A and CIP2A proteins. In addition, relative quantitation of EGFR-associated proteins by using cells with and without EGF-based EGFR activation revealed enrichment of several proteins, indicating that EGFR activation leads to its differential association with interactors. The urokinase plasminogen activator receptor (uPAR) is expressed in multiple cancers and involved in proteolytic function (*i.e.*, degradation of extracellular matrix) to promote tumor progression as well as in non-proteolytic functions (e.g., cell proliferation) [86]. The interaction partners of uPAR has been studied in ovarian cancer cells by AP-MS approach to understand its molecular functions, which uncovered five known and eight novel uPAR-associated proteins [86]. This study demonstrated the uPAR interaction with integrin β6 and lynchpin behavior of uPAR in tumor progression. The interaction partners of estrogen receptor (ER) sub types (ERα and ERβ) have been well studied by AP-MS analysis. The increased ERα activity plays a critical role in cancer by promoting proliferation and decreasing apoptosis. However, ERα and ERβ generally show opposite effects [87]. Ligand-activated ERα-associated proteins (*n* = 264) were identified in the nuclei of human breast cancer cells (MCF-7) [88]. Another study has identified 303 co-purified proteins of ERβ from nuclear extracts of MCF-7 cells [89]. Moreover, cytoplasmic protein interaction partners were uncovered by analyzing the unliganded ERα and ERβ interactomes in MCF-7 cells [90]. The interactomes of unliganded ERα and ERβ consists of 84 and 142 proteins, respectively. These studies are critical to understand the functional roles and the signaling pathways associated with ER subtypes.

By reviewing the literature, we tried to emphasize the protein complexes identified in several cancer proteomics studies as follows: (i) the protein arginine methyltransferases (PRMTs) are deregulated in multiple cancers. It is known that more than 90% of the methylation of arginine residues is controlled by the activity of PRMT1, which is linked with transcription regulation [91]. PRMT1 is not only overexpressed but also aberrantly spliced in multiple cancers [91]. To understand the splice variant-specific functions of PRMT1v1 and PRMT1v2, their interactomes have been uncovered in breast cancer cells by the AP-MS approach [49]. The interactomes of PRMT1v1 and PRMT1v2 were identified with eight and 33 specific proteins, respectively. The interactions between PRMT1v1 and PARP1, and between PRMT1v2 and MYO1B or XPO1 were validated by western blot analysis. In addition shared interactions between FUS and PRMT1v1 or PRMT1v2 were validated. This study revealed functional roles of PRMT1v1 and PRMT1v2 in agreement with their cellular localization; (ii) the high levels of tetraspanins, which are a group of integral membrane proteins, may promote tumor progression. The interactome of tetraspanin CD9 has been investigated in colon cancer cells. This study has identified 32 proteins associated with CD9 including a protein complex between CD9 and epithelial cell adhesion molecule (EpCAM) [92]; (iii) a recent study has demonstrated a knock-in AP-MS approach using epitope-tagging of endogenous genes in cancer cells. This study has explored the interaction partners of colorectal cancer-associated genes, including adenomatous polyposis coli (APC). In particular, the interactions between APC and β-catenin, FLII, ERBB2IP, or TPM1 have been validated [93]; (iv) by using TAP system and MudPIT approach, Koch and coworkers have reported the interactors of c-Myc oncoprotein in colorectal cancer cells and embryonic kidney cells [94]. This is the first in-depth study of c-Myc interactors that identified 221 interactors. Further, several c-Myc protein complexes (DBC-1, FBX29, KU70, MCM7, Mi2-b/CHD4, RNA Pol II, RFC2, RFC3, SV40 Large T Antigen, TCP1α, U5-116kD, and ZNF281) were confirmed. (v) Sox2 is well-known for its roles not only in development and maintenance of pluripotency but also in cancers [95]. By using the FLAG tag system and MudPIT, the Sox2 interactome comprising about 280 proteins has been identified in medulloblastoma cells to understand the behavior of Sox2 in brain cancer cells [96]. The complexes of two proteins (MSI2 and USP7) with Sox2 were confirmed by CoIP; (vi) the interactome of C1QBP (complement component 1, q subcomponent binding protein) has been studied by using the FLAG tag system and MS analysis [97]. A total of 187 proteins were identified as interactors within the C1QBP complex. This study has observed the elevated levels of C1QBP in breast cancer cells and showed its interaction with PRKCZ (protein kinase Cζ) is involved in chemotaxis and metastasis; and (vii) the MS-based parallel affinity purification (PAP) study has described the interaction partners of tumor suppressor PTEN (phosphatase and tensin homologue) and its cancer-associated mutant form, G20E PTEN [50]. The PAP analysis prioritized four wild-type-specific and five mutant-specific and 11 common interacting proteins. This study not only confirmed the wild-type-specific (NUDT16L1) and mutant-specific (CALU) interactors of PTEN but also the common interactors (IQGAP1 and USP7) of both types. The above examples indicates that AP-MS is a powerful approach to characterize the protein complexes in cancer cells.

## 7. Conclusions and Perspectives

AP-MS is the most widely accepted approach to characterize protein complexes of target proteins due to the robustness and sensitivity of the MS analysis. Experimental or computational approaches have linked several protein complexes with cellular processes and molecular functions by using Gene Ontology annotations [56,57,98]. This may enhance our knowledge on the physiological and pathological functional roles of proteins and their protein interactions. However, the AP-MS data does not always explain the direct and indirect interactions between a target protein and its interaction partners [14,15] and may bypass transient interactions with a single time point experiment. Experimental approaches (*in vivo*/*in vitro*), computational analysis (*in silico*), and resource interaction databases are necessary to map direct and indirect protein interactions identified through the AP-MS approach. The quantitative AP-MS analysis has been used to identify the contaminants, which show non-specific interactions with the target protein. We believe that the major applications of quantitative AP-MS approach are to compare the interactors of the protein complex under different biological conditions and to study the protein interaction dynamics. However, a relatively large number of studies have demonstrated the static interactions of target proteins (*i.e.*, normal/physiological condition, or disease condition) but not the differential interactions and protein interaction dynamics of target proteins (*i.e.*, normal *vs.* treated/diseased condition) [4,48]. It is necessary to focus on the dynamics of protein interactions of target protein, which may aid to understand the signal transduction processes linked with enzymes such as kinases and phosphatases. In addition, it is important to focus on the differential interactomes of disease-promoting genes to understand the molecular mechanisms associated with the pathogenesis.

The Encyclopedia of DNA Elements (ENCODE) project revealed 20,687 protein-coding genes in the human genome [99]. In general, mutations, alternative splice variants and posttranslational modifications are the sources of a large number of protein variants of these protein-coding genes. Understanding of the functions of each protein variant in different cell conditions is highly complicated because of the protein interactions [3]. In the future, the characterization of protein complexes of splice variants of each gene under different biological conditions is necessary. It is also necessary to store the information related to the variant specific, compartment-specific (*i.e.*, protein localization-specific), cell condition-specific (*i.e.*, healthy/diseased state-specific), and tissue-specific protein-protein interactions in databases. In addition, it is important to store the protein interaction dynamics and differential interactomes in databases. Moreover, the databases should include options to look into the above information for each gene and its variants. This may further enhance our understanding of the protein functional roles in pathogenesis as well as physiological conditions.
